# C-reactive protein is not a biomarker of depression severity in drug-naïve obese patients with metabolic syndrome

**DOI:** 10.1017/neu.2025.10034

**Published:** 2025-08-26

**Authors:** Abbas F. Almulla, Spas Kitov, Tanya Deneva, Maria-Florance Kitova, Lyudmila Kitova, Kristina Stoyanova, Drozdstoy Stoyanov, Michael Maes

**Affiliations:** 1 Sichuan Provincial Center for Mental Health, Sichuan Provincial People’s Hospital, School of Medicine, University of Electronic Science and Technology of China, Chengdu, China; 2 Key Laboratory of Psychosomatic Medicine, Chinese Academy of Medical Sciences, Chengdu, China; 3 Medical Laboratory Technology Department, College of Medical Technology, The Islamic University, Najaf, Iraq; 4 Section of Cardiology, First Department of Internal Diseases, Medical University of Plovdiv, Plovdiv, Bulgaria; 5 Clinic of Cardiology, St. George University Hospital, Plovdiv, Bulgaria; 6 Department of Clinical Laboratory, Faculty of Medicine, Medical University of Plovdiv, Plovdiv, Bulgaria; 7 5th years at Faculty of Medicine, Medical University of Plovdiv, Plovdiv, Bulgaria; 8 Research Institute at Medical University of Plovdiv, Medical University of Plovdiv, Plovdiv, Bulgaria; 9 Research and Innovation Program for the Development of MU - PLOVDIV (SRIPD-MUP), Creation of a network of research higher schools, National Plan for Recovery and Sustainability, European Union–NextGenerationEU, Medical University of Plovdiv, Plovdiv, Bulgaria; 10 Kyung Hee University, Seoul, Republic of Korea

**Keywords:** Metabolic syndrome, depression, inflammation, hsCRP, obesity

## Abstract

**Background:**

Metabolic syndrome (MetS) is highly prevalent among adults and is frequently accompanied by depressive symptoms. While high-sensitivity C-reactive protein (hsCRP) has been proposed as a potential indicator of depression, existing evidence remains inconclusive.

**Objective:**

This study aimed to determine whether increased serum hsCRP or other immune-metabolic biomarkers are associated with depressive symptoms in drug-naïve individuals with obesity and MetS.

**Methods:**

A total of 88 drug-naïve patients with obesity and MetS but without coronary artery disease were enrolled and serum levels of neuro-immune and metabolic biomarkers were assessed.

**Results:**

In MetS, the severity of depression, as assessed using the von Zerssen Depression Rating (VZDR) scale was significantly associated with interleukin (IL)-6, leukocyte numbers, triglyceride × glucose (Tyg) index, low-density lipoprotein cholesterol, Apolipoprotein B (all positively) and mean platelet volume (MPV), visfatin and adiponectin (all negatively). There were no significant associations between hsCRP and severity of depression. In MetS patients, hsCRP is strongly associated with increased leukocyte numbers, alkaline phosphatase, γ-glutamyl transferase, uric acid, platelet numbers and MPV, thereby shaping a distinct subtype of MetS, which is not related to depression.

**Conclusions:**

Our findings indicate that depressive symptoms in MetS patients are associated with immune–metabolic biomarkers indicating immune activation, atherogenicity and insulin resistance, but not with hsCRP. The reason is that hsCRP in MetS is a biomarker of a specific MetS subtype that is characterized by megakaryopoiesis, hepatocyte activation, and uric acid production, which were not associated with depression.

## Graphical abstract

**Figure:**
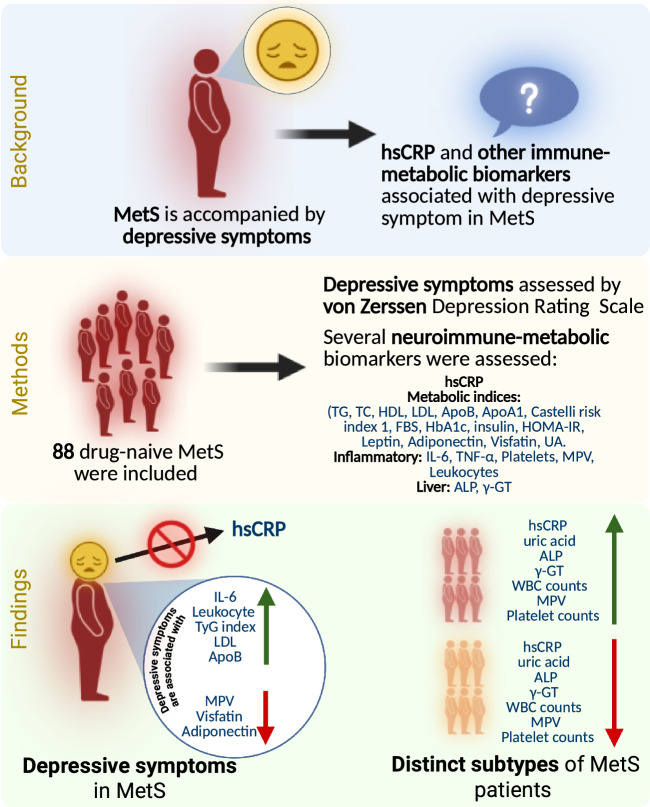

Significant Outcomes

This research indicates that hsCRP is not correlated with depressive symptoms in drug-naïve individuals diagnosed with metabolic syndrome (MetS). Depressive severity was predicted by immune activation (IL-6, leukocyte count), atherogenicity (ApoB, LDL-cholesterol), insulin resistance (TyG index), and reductions in visfatin, adiponectin, and mean platelet volume. In contrast, hsCRP identified a specific MetS phenotype, characterized by liver dysfunction (γ-GT, ALP), elevated uric acid, platelet activation, and leukocytosis, which was not associated with depression.

Limitations

- The identification of two MetS subgroups based on the LIPUR index requires replication in diverse and non-obese cohorts.
- The lack of cytokine, chemokine, and growth factor measurements limits a comprehensive interpretation of immune-inflammatory findings.

Highlights

- hsCRP showed no association with depressive symptoms in patients with metabolic syndrome (MetS).
- Depressive symptoms in patients with MetS are associated with immune and metabolic biomarkers, but not with hsCRP.
- hsCRP identifies a distinct MetS subtype with liver dysfunction, immune and platelet activation, and higher uric acid.

## Introduction

Metabolic syndrome (MetS) impacts around 20–25% of the adult population globally and represents a significant public health issue due to its correlation with central obesity, hypertension, dyslipidaemia, insulin resistance, and hyperglycemia (Nolan *et al*., 2017; Fahed *et al*., 2022), increasing the likelihood of developing coronary artery disease (Alshammary *et al*., 2021). Major depressive disorder (MDD) often coexists with MetS, leading to further adverse effects on clinical outcomes and overall quality of life (de Melo *et al*., 2017). Accumulating studies indicate that MetS may serve as a predictor of depressive symptoms, especially in middle-aged and older individuals (Akbaraly *et al*., 2009; Akbaraly *et al*., 2011). However, depressive symptoms may, in turn, induce MetS (Pulkki-Råback *et al*., 2009; Gurka *et al*., 2016).

Depressive symptoms in various conditions are linked to changes in lipid profiles, insulin resistance, hypertension, and indicators of hepatic dysfunction–pathophysiological characteristics frequently seen in individuals with MetS (Akbaraly *et al*., 2009; Zelber-Sagi *et al*., 2013; de Melo *et al*., 2017; Villarreal-Zegarra and Bernabe-Ortiz, 2020; Maes *et al*., 2023). MetS and mood disorders exhibit shared immuno-inflammatory and oxidative stress pathways, characterized by elevated atherogenic indices and diminished levels of endogenous antioxidants and anti-inflammatory mediators (de Melo *et al*., 2017).

Elevated high-sensitivity C-reactive protein (hsCRP), a well-established marker of systemic inflammation, has been consistently observed in individuals with MetS (Manoj Sigdel, 2014; Shih *et al*., 2022). Increased hsCRP levels are associated with a heightened risk of depressive symptoms including in people with metabolic or cardiovascular comorbidities (Heisey *et al*., 2024; Ji *et al*., 2024). Recent findings propose to stratify depressed patients based on their hsCRP levels as ‘inflammatory depression’ (if hsCRP ≥3 mg/L) (Wessa *et al*., 2024). However, some authors observed that the slight association between hsCRP and depressive symptoms disappeared after adjusting for body mass index (BMI), indicating that obesity may mediate or confound this relationship (Douglas *et al*., 2004; Khan *et al*., 2020). Other (Tully *et al*., 2015; Ji *et al*., 2024), but not all (Krogh *et al*., 2014) studies indicate a positive correlation between hsCRP levels and depressive symptoms. Furthermore, low serum levels of hsCRP demonstrate a lack of specificity as a biomarker for depression, given their overlap with indicators of MetS, subclinical atherosclerosis, and increased BMI (Maes, 2025).

Adipokines have garnered attention for their role in linking metabolic and mood disorders. Elevated leptin, primarily produced by adipocytes, has been consistently associated with an increase in depressive symptoms, while adiponectin typically exhibits a negative correlation (Labad *et al*., 2012; Chirinos *et al*., 2013). Moreover, leptin levels exhibit a positive correlation with hsCRP, while adiponectin levels show a negative correlation (Shamsuzzaman *et al*., 2004; Komatsu *et al*., 2007). This underscores a significant relationship between systemic inflammation and metabolic dysregulation. The intricate relationship between MetS and depressive disorders, especially via neuroimmune mechanisms, necessitates that research on immune and metabolic profiles in MDD considers stratification according to the presence of MetS (Maes *et al*., 2024). Nevertheless, the associations between depression on hsCRP and other immune-metabolic biomarkers in drug-naïve obese patients with MetS and without coronary artery disease (CAD) have remained elusive.

Hence, this study investigates the association between depressive symptoms and hsCRP and other immune-metabolic biomarkers in patients with drug-naïve MetS without CAD. The immune-metabolic biomarkers encompass lipid profiles, apolipoproteins A1 (ApoA1) and ApoB, atherogenic indices, insulin resistance biomarkers, adipokines (leptin, adiponectin, visfatin), inflammatory cytokines (TNF-α, IL-6, hsCRP), blood cell indices such as white blood cell count, platelet count, mean platelet volume (MPV), serum uric acid, and liver enzymes, i.e. gamma-glutamyl transferase (γ-GT) and alkaline phosphatase (ALP). Based on the current state-of-the-art knowledge, the specific hypothesis is that depressive symptoms in the target population are more closely linked to underlying metabolic abnormalities than to hsCRP levels alone.

## Participants and methods

### Participants

**Figure 1.**
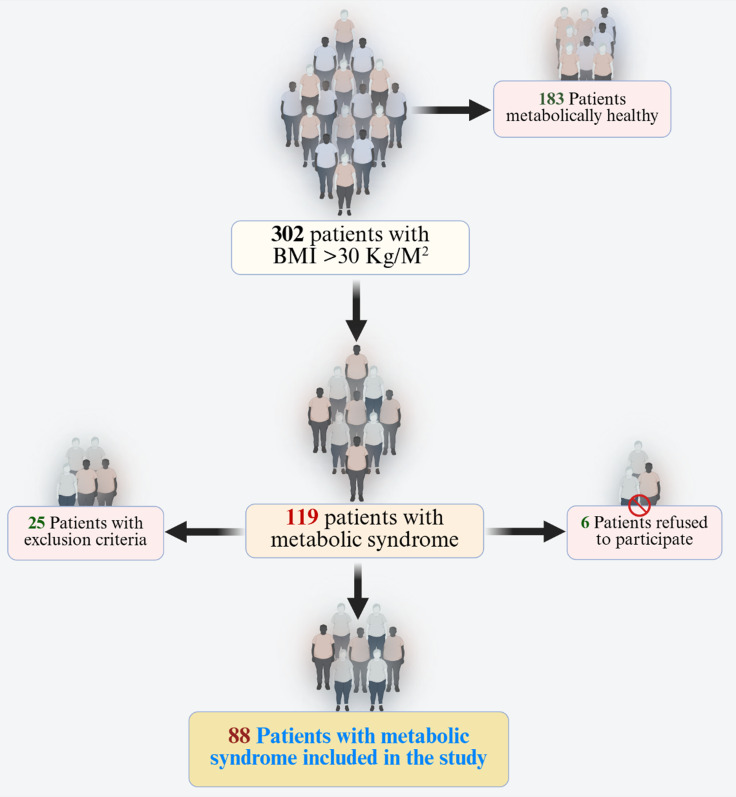
Patient selection algorithm.

Figure 1 shows the flow of the patient through the study. 302 obese patients with BMI over 30 kg/m^2^, waist circumference over 80 cm for women and 94 cm for men, aged 35–55 years and previously untreated, were screened. All underwent clinical and laboratory screening to select participants with MetS. The diagnosis was made according to the recommendations of the International Diabetes Federation (Alberti *et al*., 2009) as the presence of three or more of the following criteria: waist circumference (according to the guidelines set by the International Diabetes Federation for Europeans): waist circumference over 80 cm for women and 94 cm for men, body mass index over 30, triglycerides ≥1.7 mmol/l; HDL cholesterol <1.0 mmol/l in women or <0.9 mmo9l/l in men, blood pressure ≥130/85 mmHg or those taking medication for hypertension, fasting blood sugar ≥5.6 mmol/l or history of taking antidiabetic medication. Ischemic heart disease was excluded in all patients with exercise stress testing, CT angiography, or selective coronary angiography according to European Guidelines (Vrints *et al*., 2024).

Other exclusion criteria were chronic renal failure, chronic liver disorders, chronic lung disease, moderate or severe valvular heart disease, congenital heart disease, left ventricular systolic dysfunction on echocardiography, pregnancy, known malignancy, thyroid disease, electrolyte imbalance, and conduction disorders, as well as patients taking medications known to affect the ECG repolarization parameters and patients who have had an infection less than 2 weeks ago. We also excluded subjects with neuroinflammatory or neurodegenerative disease, immune disorders, including inflammatory bowel disease, COPD, and rheumatoid arthritis, and those with lifetime major psychiatric disorders. During the selection of suitable patients, 25 patients were excluded due to concomitant CHD or refusal to participate in the study, and 6 refused to participate. Finally, a group of 88 patients with newly diagnosed metabolic syndrome aged 35–55 years and without concomitant CHD was recruited.

### Clinical assessments

#### Adiposity-related markers

Using the InBody_270_ analyzer (InBody Co., Ltd, Korea) we assessed anthropometric measurements including body weight, height, body mass index (BMI, i.e. weight in kilograms divided by height in meters squared), waist-to-hip ratio (WHR, and total body fat percentage. Waist circumference was measured at the level of the umbilicus. Waist-to-hip ratio was calculated from waist circumference measured at the midway between the last palpable rib and the iliac crest and hip circumference (World Health, 2011). Body fat percent was estimated based on multifrequency bioelectrical impedance analysis. The latter technique uses varying frequencies of alternating current that are sent through the body, where passing through different tissues alters the resulting voltage, which information is later used to predict BF%. This renders reasonable estimates compared with the reference method dual-energy X-ray absorptiometry (McLester *et al*., 2020). Blood pressure was measured early in the morning, after 10 minutes of rest in a quiet room, while subjects were in a sitting position and using a validated oscillometric device with an appropriately sized cuff applied to the right upper arm. The average of three blood pressure readings (obtained at 1-minute intervals) was taken as the final result.

To assess severity of depression, we used the von Zerssen Depression Rating (VZDR) Scale (Krastev, 2020), translated into Bulgarian. Nevertheless, using principal component analysis, it appeared that no general factor could be extracted from the 16 scale item scores. In fact, the scree plot showed that 5 PCs could be extracted with eigenvalues >1 explaining only 58.8% of the variance. As such, the total sum on this rating scale does not reflect the total severity of illness. We found that one PC could be extracted from 6 items, which explained 52.2% of the variance, whereby all 6 items showed higher loadings (>0.7). These items are ‘I am afraid of losing my mind’, ‘I feel melancholic and depressed’, ‘I would like to take my life’, ‘I often feel simply miserable’, ‘I cannot think straight’, and ‘I don’t have any feelings anymore’. These items are indeed indicative of a more severe depression phenotype. Therefore, we used the sum on those 6 items as an index of severity of depression (labeled as VZDR6) together with the total sum (VZDRtotal).

#### Blood biomarker assays

Fasting venous blood samples were collected in accordance with standard procedures and serum from each participant was aliquoted and stored at −20 ˚C until analysis, with a maximum storage time of two months.

Table 1 presents the reference values of the indicators tested in the Central Institute Clinical Laboratory of MU-Plovdiv, Plovdiv, Bulgaria. Hematological analyses, i.e., number of leukocytes and platelets, mean platelet volume (MPV) were performed using the automatic hematological analyzer Advia 2120i using the original reagents from the manufacturer (Siemens Healthcare, Germany). The intra-assay coefficient of variation (CV) for all analytes was between 0.67% and 2.96%, and the inter-assay CV values were between 0.93% to 3.39%.

**Table 1.** The biomarkers examined in the study and their reference values

Biomarkers	Units	Reference range in our laboratory
1. Immune biomarkers		
Interleukin (IL)-6	pg/ml	≤ 17
Tumor necrosis factor (TNF)-α	pg/ml	<12.4
High sensitivity C-reactive protein (hsCRP)	mg/L	<3: normal >3-10: normal or slightly elevated (obesity, MetS, minor infection) >10-100: moderate inflammation, immune disorders >100: acute bacterial and viral infections
2. Hematology		
Number leukocytes	10 ^9^/ l	3.5–10.5
Number platelets	10 ^9^/ l	140–400
Mean platelet volume (MPV)	fL	7.5–11.5
3. Insulin resistance and atherogenicity biomarkers		
Fasting blood glucose (FBG)	mmol/l	2.8–6.1
Immunoreactive insulin	mIU/l	1.9–23
HOMA index = FBG x insulin/22.4		≤ 2.5
Glycated hemoglobin (HbA1c)	%	4.5–6.3
Total cholesterol (TC)	mmol/l	3.0–5.2
Triglycerides (TG)	mmol/l	0.4–1.7
High-density lipoprotein-cholesterol (HDL-C)	mmol/l	≥0.9 male >1.15 female
LDL-cholesterol	mmol/l	<1.8 mmol/l high CV risk <2.6 mmol/l moderate CV risk
TC/HDL-C ratio (Castelli risk index1)	-	≥ 5: increased risk
TyG index = TG x FBG / 2		4-8
Аpolipoprotein-В (ApoB)	g/l	0.63–1.88 male 0.56–1.82 female
Аpolipoprotein-А1 (ApoA1)	g/l	1.09–1.84 male 1.06–2.28 female
4. Adipokines		
Adiponectin	mg/mL	5-30
Leptin	ng/ml	3,7–11,1 female 2,0–5,6 male
Visfatin	ng/mL	0.2–1.5
5. Biochemistry		
Uric acid	µmol/l	208–387 male 149–363 female
Alkaline phosphatase	U/l	30–120
γ-glutamyl transferase	U/l	0–55 male 0–35 female

CV: cardio-vascular risk.

Biochemical parameters (blood glucose, LDL-cholesterol, total cholesterol, HDL-cholesterol, triglycerides, Apo A1, Apo B, uric acid, HbA1c, alkaline phosphatase, γ-GT) were determined using standardized methods recommended by the International Federation of Clinical Chemistry and Laboratory Medicine (IFCC). The tests were carried out using Beckman Coulter reagents on an automated clinical chemistry analyzer Olympus AU 480 (Beckman Coulter, Inc., USA) according to original programs. The analytical intra-assay reliability for all indicators was between 0.37% and 1.06%, and the inter-assay CV was between 0.67% and 2.19%. Serum hsCRP levels were measured by immunoturbidimetry with latex agglutination using an AU 480 Beckman Coulter clinical chemistry analyzer (Olympus AU 480, Beckman Coulter, Inc., USA). The intra-assay and inter-assay CV values of this assay were < 4.87%. Serum insulin was assayed using a one-step immunoenzymatic sandwich method based on a chemiluminescent principle c (CLIA) on the Access 2 Immunoassay System (Beckman Coulter, Inc., USA). The intra-assay and inter-assay CV values of the insulin assay were < 4.2%. The serum concentrations of TNF-α, IL-6, leptin (LDN, Germany), visfatin, and high-sensitivity adiponectin were determined using competitive enzyme-linked immunosorbent assays (BioVendor, USA), following validation at the local level. The methods show high precision with intra-assay CV<10% and inter-assay CV <12%.

Consequently, we computed 6 different indices, namely, Castell risk index 1 as total cholesterol / HDL and the ApoB / ApoA1 ratio, which are both pro-atherogenic indices; LDL / ApoB or LAR index, reflecting the size and composition of LDL particles with smaller particles being more pro-atherogenic; the HOMA-IR index, which estimates insulin resistance; the TyG index = Ln [fasting triglycerides (mg/dL) x fasting blood glucose (mg/dL)/2), which is a surrogate marker of insulin resistance; and the adiponectin / leptin ratio as a marker of dysfunctional adipose tissue (Frühbeck *et al*., 2019).

#### Data analysis

In the current study we employed IBM SPSS Statistics for Windows Version 30 to conduct all statistical analyses. Differences in continuous variables across groups were assessed using analysis of variance (ANOVA), while analysis of contingency tables using the chi-square (χ^2^) test was utilized to investigate relationships among categorical variables. Bivariate relationships among continuous variables were examined utilizing Pearson’s product-moment correlation coefficients. Multiple comparisons were adjusted for False-Discovery Rate (FDR) p-correction. Multivariate regression analyses were performed to identify predictors of the severity of depression. A manual and stepwise approach was employed to determine the most significant explanatory variables and generate partial regression plots. Key assumptions, including multivariate normality, homoscedasticity, and the absence of collinearity and multicollinearity, were verified prior to constructing the final regression models. A significant threshold of *p* < 0.05 was utilized for all statistical tests, employing a two-tailed methodology.

Principal component analysis (PCA) was employed to extract latent constructs from the clinical items and biomarker sets. The Kaiser–Meyer–Olkin (KMO) statistic was employed to assess sampling adequacy, with values exceeding 0.7 indicating sufficient factorability. A principal component is considered valid when the explained variance surpasses 50%, Cronbach’s alpha for internal consistency exceeds 0.70, and all factor loadings on the first component are greater than 0.7. A two-step cluster analysis was employed to generate clusters of patients based on biomarker values. The silhouette coefficient of cohesion and separation was used to assess the quality of the cluster solution, with values exceeding 0.5 indicating adequate cohesion and separation.

The primary statistical analysis is the multiple regression analysis with severity of depression scores as outcome variables and up to 5 covariates. Power analysis showed that when using an effect size of 0.25, *p* = 0.05, power = 0.8, and max 5 predictors, the minimum number of subjects should be *n* = 58.

## Results

### Socio-demographic and clinical characteristics of the study group

**Figure 2.**
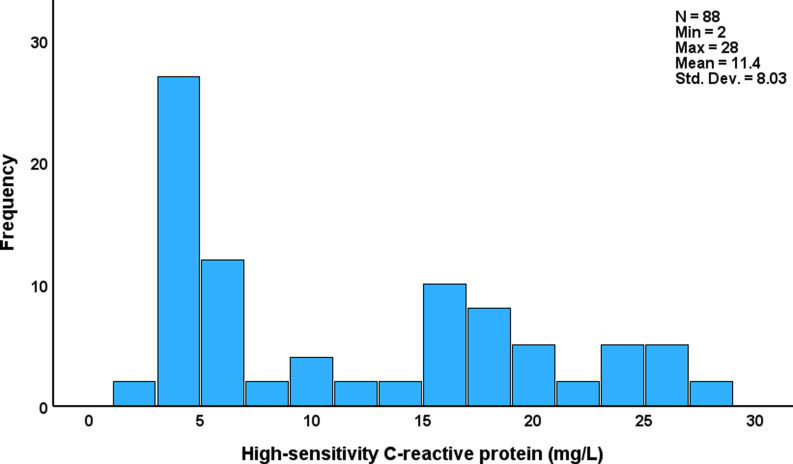
Distribution of high-sensitivity C-reactive protein serum levels in patients with metabolic syndrome and obesity.

Fig. 2 shows the bimodal data distribution of the hsCRP data. Visual binning revealed that a hsCRP value >13.5 mg/L could be used to dichotomize the patient group into 2 subgroups, namely those with low (n = 50) versus high (n-38) hsCRP levels. Table 2 compares the socio-demographic and clinical variables among MetS patients categorized by hsCRP levels, utilizing a cutoff of 13.5 mg/L. No statistically significant differences were found between the both high-hsCRP and low-hsCRP groups for all variables assessed.

**Table 2.** Demographic and clinical data of patients with metabolic syndrome (MetS) and obesity with higher versus lower high-sensitivity C-reactive protein (hsCRP) values (13.5 mg/dL cutoff)

Variables	MetS Patients with lower hsCRP (n = 50)	MetS Patients with higher hsCRP (n = 38)	F / χ^2^	df	*p*-value
Gender (F/M)	23/27	14/24	0.74	1	0.513
Smoking (Yes/No)	18/32	19/19	1.74	1	0.200
Cigarette pack‑years	22.6 (12.1)	19.5 (7.3)	0.93	1/42	0.340
Body Mass Index (kg/m^2^)	40.16 (9.89)	40.06 (5.91)	0.00	1/86	0.955
Waist circumference (cm)	117.2 (22.2)	113.2 (19.9)	0.78	1/86	0.380
Waist‑hip ratio	1.087 (0.100)	1.107 (0.091)	0.93	1/86	0.337
Body fat (%)	38.51 (8.41)	38.79 (7.49)	0.03	1/86	0.869
Arterial pressure (no/high normal - 135–139/85–89/stage 1 - 140–160/90–100/stage 2 - > 160/100	7/18/16/6	2/9/14/10	5.27	–	0.153

Results are shown as mean (SD) or as ratios.

### Depression scores and biomarkers in the high versus low-hsCRP groups

Table 3 compares the biomarkers among MetS patients dichotomized by hsCRP levels, utilizing a cutoff of 13.5 mg/L. No significant differences were found between the groups regarding both the VZDRtotal and VZDR6 scores. Moreover, there were no differences in serum triglycerides, total cholesterol, HDL, LDL, Castelli Risk Index 1, ApoB/ApoA1 ratio, LAR ratio, apolipoprotein B, apolipoprotein A1, fasting blood glucose, HbA1c, immunoreactive insulin, HOMA-IR, TyG index, leptin, adiponectin, visfatin, IL-6, or TNF-α between both hsCRP groups (even without p-correction). Nevertheless, patients allocated to the high-hsCRP group demonstrated markedly elevated levels of platelet counts, MPV, WBC count, uric acid, ALP, and γ-GT in comparison to individuals with lower hsCRP levels. These differences remained significant after FDR p-correction (at *p* = 0.0034). As a consequence, we have investigated whether one validated PC could be extracted from the hsCRP, platelet counts, MPV, WBC count, uric acid, ALP, and γ-GT data.

**Table 3.** Biomarkers in metabolic syndrome (MetS) patients with higher versus lower high-sensitivity C-reactive protein (hsCRP) values (13.5 mg/dL cutoff)

Variables	MetS Patients with lower hsCRP (n = 50)	MetS Patients with higher hsCRP (n = 38)	F	df	*p*-value
hsCRP (mg/L)	5.06 (2.56)	19.76 (4.08)	429.00	1/86	<0.001
VZDR6	11.3 (2.2)	10.3 (2.2)	3.65	1/86	0.059
VZDRtotal	28.3 (5.8)	27.5 (5.9)	0.36	1/86	0.550
Triglycerides (mmol/L)	2.75 (1.63)	2.36 (1.16)	1.55	1/86	0.216
Total cholesterol (mmol/L)	5.84 (1.23)	5.74 (1.04)	0.15	1/86	0.699
HDL‑C (mmol/L)	1.15 (0.27)	1.20 (0.29)	0.63	1/86	0.431
LDL‑C (mmol/L)	3.50 (1.15)	3.59 (0.76)	0.16	1/86	0.692
Apo B (g/L)	1.50 (0.48)	1.51 (0.65)	0.02	1/86	0.900
Apo A1 (g/L)	1.39 (0.39)	1.48 (0.41)	0.96	1/86	0.329
Castelli risk index 1	5.27 (1.49)	4.96 (1.12)	1.21	1/86	0.275
ApoB/ApoA1	1.21 (0.63)	1.19 (0.84)	0.10	1/86	0.922
LAR index (LDL/ApoB)	2.56 (1.14)	2.79 (1.20)	0.87	1/86	0.353
Fasting blood glucose (mmol/L)	5.84 (1.38)	6.03 (1.13)	0.46	1/86	0.501
HbA1c (%)	5.87 (0.64)	5.67 (0.77)	1.81	1/86	0.182
Immunoreactive insulin (µIU/mL)	23.79 (14.16)	23.05 (17.61)	0.05	1/86	0.826
HOMA‑IR	6.41 (4.53)	6.58 (6.63)	0.02	1/86	0.890
TyG index	0.46 (0.30)	0.39 (0.22)	1.60	1/86	0.209
Leptin (ng/mL)	64.94 (53.97)	68.20 (53.79)	0.08	1/86	0.779
Adiponectin (µg/mL)	7.76 (3.88)	7.98 (3.01)	0.09	1/86	0.771
Adiponectin/leptin ratio*	1.33 (7.61)	0.39 (1.11)	0.10	1/86	0.920
Visfatin (ng/mL)	5.74 (2.99)	6.06 (2.52)	0.30	1/86	0.587
IL-6 (pg/mL)	45.05 (92.98)	53.17 (86.51)	0.17	1/86	0.677
TNF‑α (pg/mL)	14.29 (27.18)	19.11 (10.86)	1.07	1/86	0.305
Platelets (×10^9^/L)	268.46 (80.78)	394.63 (59.85)	65.36	1/86	<0.001
MPV (fL)	9.50 (2.97)	14.42 (2.97)	59.18	1/86	<0.001
Leukocytes (×10^9^/L)	6.68 (2.82)	10.49 (2.18)	46.76	1/85	<0.001
Uric acid (µmol/L)	322.60 (85.21)	422.74 (41.30)	44.45	1/86	<0.001
ALP (U/L)	177.96 (86.69)	266.74 (68.47)	27.02	1/86	<0.001
γ‑GT (U/L)	59.48 (22.19)	82.32 (23.01)	22.14	1/86	<0.001
LIPUR index (z score)	−0.62 (0.76)	0.84 (0.60)	93.35	1/85	<0.001

* Processed in Log transformation; hsCRP: high-sensitivity C-reactive protein, HDL-C: high-density lipoprotein cholesterol, LDL-C: low-density lipoprotein cholesterol, Apo: Apolipoprotein, HbA1c: glycated hemoglobin, HOMA‑IR: Homeostatic Model Assessment for Insulin Resistance, TG: Triglycerides, IL: interleukin, TNF: tumor necrosis factor- α, MPV: Mean platelet volume, WBCs: white blood cells, ALP: Alkaline phosphatase, γ‑GT: Gamma-glutamyl transferase, LIPUR index: principal component extracted from hsCRP, platelets, MPV, WBCs, Uric acid, ALP, and γ‑GT.

### Results of PCA

As shown in Table 4, PCA produced one PC1 that explained 65.67% of the total variance in the hsCRP, platelet count, MPV, WBC count, uric acid, ALP, and γ-GT data. All included variables showed factor loadings exceeding 0.70, and the component displayed satisfactory internal consistency, as evidenced by an adequate Cronbach’s alpha value. This PC score was designated as the LIPUR index, reflecting its integration of biomarkers representing liver dysfunction (GGT, ALP), immune activation (hsCRP and leukocytes), platelet activity (platelet count and MPV), and uric acid metabolism.

**Table 4.** Results of Principal Component Analyses (PCA) performed on the biomarkers that are increased in patients with increased high sensitivity C-reactive protein (hsCRP) values

Variables	Loadings
hsCRP	0.705
Platelets	0.866
MPV	0.863
WBCs	0.842
UA	0.786
ALP	0.778
γ-GT	0.820
KMO	0.925
χ2	470.25
df	21
p	<0.001
Variance explained	65.67%
Cronbach’s alpha	0.92

Abbreviations: KMO, Kaiser-Meyer-Olkin measure of sampling adequacy; χ2, Bartlett’s test; df, degree of freedom; p, p-value; VE, variance explained; MPV: Mean platelet volume, UA: Uric acid, ALP: Alkaline phosphatase, γ-GT: Gamma-glutamyl transferase.

**Figure 3.**
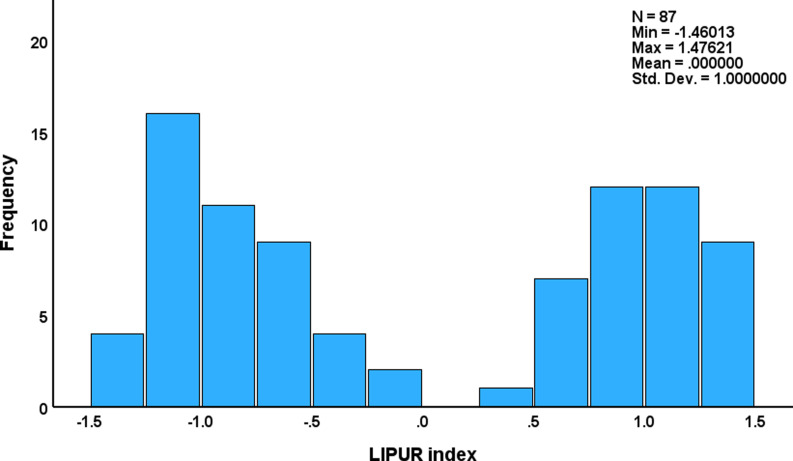
Distribution of the LIPUR index in patients with metabolic syndrome and obesity. The LIPUR index is computed as the first principal component extracted from liver tests, immune and platelet biomarkers, and uric acid.

Consequently, we computed the first PC score and displayed its distribution (see Fig. 3). This distribution was clearly bimodal. In addition, we performed a two-step cluster analysis using hsCRP, platelet counts, MPV, WBC count, uric acid, ALP, and γ-GT data as variables. This cluster analysis detected two different clusters of patients which were separated with a silhouette measure of cohesion and separation of 0.7 (more than adequate). This separation into those with high (n = 41) versus low (n = 46) LIPUR indices coincides with a threshold value of 0.0 applied to the LIPUR index. The latter was significantly higher in MetS patients with high hsCRP versus those with low hsCRP (see Table 3).

As a consequence, we have computed correlations between severity of depression and the biomarkers in all patients combined and in addition in the two distinct MetS subgroups. The latter distinction is important as there may be qualitative differences in metabolic pathways between both groups which could affect those associations.

### Correlations depression scores and biomarkers

Table 5 displays the results of multivariate regression analyses examining the relationship between both VZDR scores and the biomarkers in all subjects and in the cluster with low and high LIPUR index, while allowing for the effects of age, sex, BMI, waist-hip ratio, fat mass (%), and smoking status.

**Table 5.** Results of multiple regression analyses with the von Zerssen Depression Severity Rating (VZDR) scores as dependent variables and immune-metabolic biomarkers as explanatory variables

Dependent Variables	Explanatory Variables	Coefficients of input variables			Model statistics			
		β	t	p	R^2^	F	df	p
#1a. VZDRotal in all subjects	Model IL-6 MPV Leukocytes TyG	0.208 −0.461 0.350 0.224	2.098 −3.39 2.57 2.26	0.039 0.001 0.012 0.027	0.198	5.07	4/82	<0.001
#1b. VZDR6 in all subjects	Model MPV IL-6 WBCs TyG index Visfatin	−0.503 0.188 0.343 0.293 −0.216	−3.87 1.99 2.63 3.05 −2.25	<0.001 0.050 0.010 0.003 0.027	0.276	6.16	5/81	<0.001
#2a. VZDRtotal in subjects with low LIPUR index	Model LDL cholesterol	0.316	2.21	0.033	0.100	4.75	2/43	0.033
#2b. VZDR6 in subjects with low LIPUR index	Model Visfatin ApoB	−0.452 0.340	−3.51 2.63	0.001 0.012	0.291	8.80	2/43	<0.001
#3a. VZDRtotal in subjects with high LIPUR index	Model Adiponectin	−0.377	−2.54	0.015	0.142	6.45	1/39	0.015
#3b. VZDR6 in subjects with high LIPUR index	Model MPV TyG index	−0.395 0.328	−2.755 2.285	0.009 0.028	0.230	5.69	2/38	0.007
#4 LIPUR index in subjects with the high LIPUR cluster	Model IL-6	0.382	2.874	0.007	0.182	3.00	1/39	0.007

LIPUR index: integrated index of Liver + Uric acid + Platelet + Inflammatory biomarkers, MPV: Mean platelet volume, WBCs: White blood cells count, TG: Triglyceride, Apo: Apolipoprotein, TyG: triglyceride × fasting blood glucose index.

**Figure 4.**
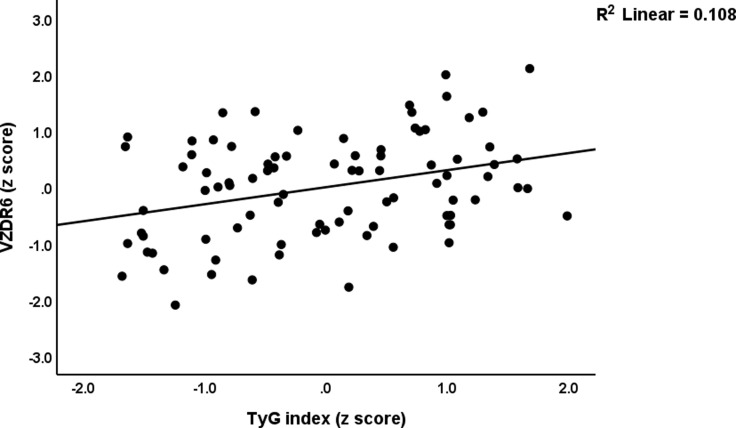
Partial regression of the von Zerssen Depression Rating (VZDR6) scale score on the TyG index (p < 0.01).

In the total study sample (regression #1a and #1b), 19.8% of the variance in the VZDRtotal score was explained by the regression on IL-6, TyG, and leukocytes (positively) and MPV (inversely). 27.6% of the variance in the VZDR6 score was explained by five significant predictors, namely MPV and visfatin (both inversely associated) and IL-6, WBC count, and TyG ratio (positively associated). Fig. 4 shows the partial regression of VZDR6 score on the TyG index.

**Figure 5.**
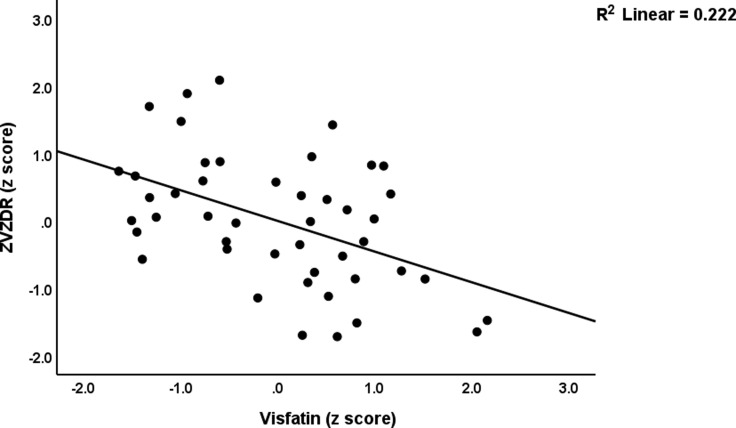
Partial regression plot illustrating the relationship between the subdomain of the von Zerssen Depression Rating (VZDR6) scale score on serum visfatin (p < 0.01).

**Figure 6.**
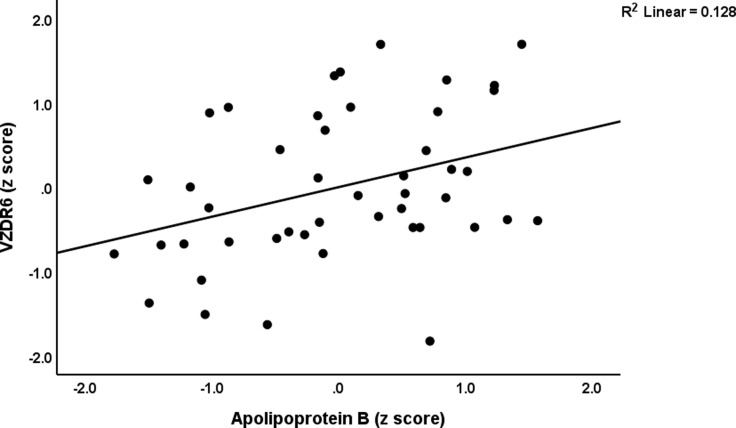
Partial regression plot illustrating the relationship between the subdomain of the von Zerssen Depression Rating (VZDR6) scale score on serum apolipoprotein B (p < 0.01).

In the low LIPUR subgroup (regression #2a and #2b), 10.0% of the variance in VZDRtotal score was explained by LDL (positively associated). 29.1% of the variance in the VZDR6 score was accounted for by visfatin (negatively associated) and apolipoprotein B (positively associated). Figures 5 and 6 show the partial regression plots illustrating the adjusted relationship between the VZDR6 score and visfatin and ApoB, respectively. In the high LIPUR subgroup (regressions #3a and #3b), lower adiponectin was associated with the VZDRtotal score explaining 14.2% of the variance. MPV was negatively associated, while the TyG ratio was positively associated with the VZDR6 score. These two variables jointly explained a significant proportion (23%) of the variance in this score. Additional regression analysis (regression #4) indicated that 18.2% of the variance in the LIPUR index in the high LIPUR cluster could significantly be predicted by IL-6.

### Depression scores and biomarkers in the LIPUR subclasses

There were no significant differences in the VZDR6 (F = 2.00, df = 1/85, *p* = 0.161) and VZDRtotal (F = 0.88, df = 1/85, *p* = 0.350) scores between patients with a lower LIPUR index and those with a higher index.

Table 6 summarizes the correlation analysis between the LIPUR index and depression severity, immune, and metabolic scores. In both the low- and high-LIPUR index clusters, no significant correlations were observed between the LIPUR indices and the depression severity scores.

**Table 6.** Intercorrelation matrix (Pearson’s correlation coefficients) between the LIPUR index and different immune-metabolic biomarkers in both clusters of metabolic syndrome patients

Variables	LIPUR index (in the low-LIPUR cluster, n = 46)	LIPUR index (in the high-LIPUR cluster, n = 41)
VZDR6	−0.124 (0.411)	−0.125 (0.438)
VZDRtotal	0.078 (0.604)	−0.063 (0.694)
Interleukin-6	−0.122 (0.417)	0.386*( 0.013)
Tumor necrosis factor-α	0.127 (0.400)	0.342* (0.029)
Fasting blood glucose	0.177 (0.239)	0.340* (0.030)
Immunoreactive insulin	0.127 (0.401)	0.343* (0.028)
HOMA-IR	0.211 (0.160)	0.341* (0.029)
Glycated hemoglobin	0.248 (0.097)	0.315* (0.045)

LIPUR index: principal component extracted from high sensitivity C-reactive protein (hsCRP), platelet number, mean platelet volume, number of leukocytes, uric acid, alkaline phosphatase, gamma-glutamyl transferase, HOMA‑IR: Homeostatic Model Assessment for Insulin Resistance, IL: interleukin, TNF: tumor necrosis factor.

In both patient clusters, there were no significant correlations between the LIPUR index and any of the atherogenic data. In patients allocated to the high LIPUR cluster, but not in those allocated to the other cluster, we found significant associations between the LIPUR index and both cytokines and insulin resistance data.

## Discussion

### Depressive symptoms in metS

The first major finding of this study indicates that the severity of depressive symptoms measured using the VZDR scale scores did not associate with increased hsCRP levels. The findings are consistent with earlier studies that did not demonstrate a reliable association between hsCRP and depressive symptoms (Mommersteeg *et al*., 2014; Krogh *et al*., 2014; Khan *et al*., 2020).

In contrast to the lack of associations between hsCRP and depressive symptoms, this study observed that severity of depression correlated mainly with immune activation (indicated by increased IL-6 and leukocyte number) and aberrations in platelets (lower MPV), atherogenicity (increased ApoB and LDL), insulin resistance (increased Ty G ratio), and lower visfatin and adiponectin.

IL-6, which induces CRP production, and increased numbers of leukocytes were associated with depressive symptoms, although the effect size of IL-6 was rather small. It is well known that depression is associated with increased serum levels of IL-6 (Maes *et al*., 1995; Osimo *et al*., 2020) and with increased numbers of leukocytes (Maes *et al*., 1993b; Foley *et al*., 2023). Depression is characterized by dysregulation in neuro-immune–metabolic–oxidative (NIMETOX) pathways (Maes *et al*., 2025a; Maes *et al*., 2025b). Jirakran *et al*., (2025b) recently reported that lipid disturbances in MDD, characterized by reduced reverse cholesterol transport and heightened atherogenicity, cannot be effectively assessed in the presence of MetS (Jirakran *et al*., 2025*b*). Recent meta-analyses found that mood disorders, including MDD and bipolar disorder, are associated with increased atherogenic indices such as the Castelli Risk Index-2, the Atherogenic Index of Plasma (Almulla *et al*., 2023; Jirakran *et al*., 2025a). Recently, Maes et al., (2024) introduced the concept of an ‘atherommune-phenome’ in outpatient MDD patients, highlighting the critical role of heightened atherogenicity linked to immune-mediated neurotoxicity in depressive pathophysiology. The alteration between MetS and MDD aggravates cytokines production and worsening symptoms (Maes *et al*., 2024). Moreover, increased insulin resistance indices contribute to the severity of depression in patients with major depression (Maes *et al*., 2025a; Maes *et al*., 2025b).

There is now some evidence that depression is accompanied by lower serum levels of adiponectin (Hu *et al*., 2015; Islam *et al*., 2022). This hormone, synthesized by adipose tissue, exhibits antioxidant, anti-inflammatory, and anti-atherogenic properties that promote metabolic and cardiovascular health (Ohashi *et al*., 2012). Experimental studies show that exogenous adiponectin produces antidepressant-like effects in animal models of stress-induced depression, indicating a potential role in mood regulation (Liu *et al*., 2012). Additionally, baseline adiponectin levels affect the effectiveness of different antidepressant combination therapies in MDD (Furman *et al*., 2018). Adiponectin is increasingly recognized as a potential therapeutic target in central nervous system disorders, including depression, owing to its neuroprotective and mood-regulating properties (Bloemer *et al*., 2018).

Visfatin is a proinflammatory adipokine that is secreted by visceral fat and is involved in insulin signaling and resistance (Alghasham and Barakat, 2008; Stofkova, 2010). Higher visfatin levels may be detected in depression (Akinlade *et al*., 2020) and the remission phase of inflammatory disease like rheumatoid arthritis or inflammatory bowel disease (Lee and Bae, 2018; Saadoun *et al*., 2021). Nevertheless, increased insulin levels might lower visfatin production (Kowalska *et al*., 2013). Importantly, visfatin (and other adipokines) has neuroprotective effects via enhancing cellular energy metabolism, and it may have glucose-lowering and insulin-enhancing effects (Fukuhara *et al*., 2005; Erfani *et al*., 2015; Lee *et al*., 2019). Thus, while increased visfatin is a marker of obesity and MetS that may aggravate lipid aberrations, atherosclerosis, and type 2 diabetes mellitus, lower levels may reduce neuroprotection, thereby possibly aggravating the pathophysiology of depression.

Lower MPV values have been found in some, but not all studies, in depression particularly following treatment (Öztürk *et al*., 2019). One explanation is that IL-6 and glucocorticoid levels, which are both increased in depression (Maes *et al*., 1993*a*) could have caused lower MPV values (Clarke *et al*., 1996). Nevertheless, in the current study, many MetS patients (those belonging to the high LIPUR cluster) display high MPV values. As such, the lowered MPV levels in our MetS patients may be regarded as a secondary phenomenon not directly related to the pathophysiology of depression.

Overall, our findings indicate that increasing severity of depression in our drug-free MetS patients is an indicant of immune activation, atherogenicity, insulin resistance, and lowered antioxidant defenses. Previous studies have repeatedly emphasized the association between particular depressive symptom profiles and components of MetS. Suttajit and Pilakanta revealed that symptoms including depressed mood, insomnia, and psychomotor retardation are significantly linked to central obesity and hyperglycemia, indicating that specific depressive phenotypes may demonstrate stronger metabolic associations (Suttajit and Pilakanta, 2013). Chirinos *et al*. (2017) identified four distinct profiles of depressive symptoms among healthy mid-life adults in the United States, each linked to different cardiometabolic outcomes (Chirinos *et al*., 2017).

Therefore, the issue remains: why is hsCRP not a biomarker of depression in MetS, whereas IL-6, which induces CRP production, is associated with depression? The following section will address this matter.

### Distinct subgroups of MetS patients

The second major finding of this study is that MetS in obese patients comprises distinct subclasses. Thus, PCA and cluster analysis revealed two biologically significant clusters based on higher and interrelated levels of hsCRP, uric acid, ALP, γ-GT, WBC counts, MPV, and platelet counts.

Mild to moderate inflammation frequently results in an increase in the number of platelets and an increase in MPV (Thomas and Storey, 2015; Korniluk *et al*., 2019). Thrombocytosis may be induced by IL-6 through an increase in thrombopoietin production (Kaser *et al*., 2001). Furthermore, megakaryopoiesis induced by IL-6 or TNF-α may also result in an increase in MPV, which is characterized by activated, larger, and younger platelets (Gardner and Bessman, 1983; Dan *et al*., 2009). Metabolic or hepatic inflammation may be indicated by elevated ALP and γ-GT levels. For instance, IL-6 may elicit both enzymes by promoting the acute phase response and activating hepatocytes (Toth *et al*., 2004; Schmidt-Arras and Rose-John, 2016). The production of uric acid can also be induced by IL-6 through stimulation of xanthine oxidase (Pfeffer *et al*., 1994) and impaired renal excretion of uric acid (Tsutani *et al*., 2000; Zhang *et al*., 2024). Intertwined associations between these LIPUR pathways and pro-inflammatory cytokines (IL-6, TNF-α) and insulin resistance indices (HOMA-IR and TyG indices and HbA1c) further characterize this particular cohort of MetS patients. As a result, the cluster of MetS patients with high LIPUR indices is distinguished by a substantially increased inflammatory load, which is induced by pro-inflammatory cytokines, as well as elevated liver enzymes, uric acid levels, and heightened immune and platelet activation.

It is important to emphasize that the subgroup with a lower LIPUR index also demonstrates substantial immune-metabolic abnormalities, specifically elevated hsCRP and IL-6 levels, HOMA-IR, TyG, Castelli risk index 1, and liver tests in comparison to the normal values (refer to Table 1). However, hsCRP is a biomarker of a specific immune-metabolic subtype of MetS, identified as the high-LIPUR subclass. This subclass is not associated with depression. Depression is more closely linked to other immune-metabolic pathways, including insulin resistance and atherogenicity, as well as diminished antioxidant defenses.

The results of the current study emphasize that, in MetS, elevated serum hsCRP levels cannot be used as a biomarker of inflammation in association with depression. Indeed, increased hsCRP is a marker of a specific subgroup of MetS, characterized by specific immune-metabolic LIPUR alterations. In depression, approximately 50% of the variance in hsCRP can be attributed to factors that are not associated with mood disorders, such as an elevated BMI and advanced age (Moraes *et al*., 2017). Based on abdominal adiposity, hyperglycemia, and BMI, Junqueira et al. demonstrated that CRP is a reliable indicator of MetS (Junqueira *et al*., 2009). Cardiovascular risk is frequently indicated by even minor increases in hsCRP within lower ranges, which may result from conditions such as obesity, subclinical atherosclerosis, or metabolic syndrome (Blaha *et al*., 2011; Timpson *et al*., 2011). Given that hsCRP is widely recognized as a marker of the metabolic-inflammatory burden associated with MetS (Mirhafez *et al*., 2016; Maes, 2025), our results substantiate the assumption that elevated hsCRP primarily indicates metabolic disturbances that are inherent to MetS, and in particular for one MetS subtype (the high LIPUR subgroup), rather than serving as a specific marker for depression.

Future research investigating the role of IL-6 in MDD and MetS may contribute to further clarifying the results of this study. IL-6 is strongly associated with the induction of Janus Kinase (JAK) / signal transducer and activator of transcription (STAT)3 pathway, with STAT3 increased in MDD (Aldossary *et al*., 2024) and MetS (Wang *et al*., 2025). As both MDD (Xu *et al*., 2024) and MetS (Mobeen *et al*., 2025) are associated with increased nuclear factor-kB, recent work indicating the interactions of STAT3 with nuclear factor-kB dimer components may be of relevance, including in the regulation of the melatonergic pathway (Córdoba-Moreno *et al*., 2024). The investigation of IL-STAT3 interactions with nuclear factor-kB dimer components will be an important topic for future investigation in MDD and MetS, as well as in wider medical conditions (Anderson, 2025).

## Limitations

It is essential to acknowledge some limitations while interpreting the current findings. The existence of two different MetS subgroups with respect to the LIPUR index deserves replication in other countries and cultures and in a cohort of MetS patients without obesity. The present study would be even more interesting if we could examine additional cytokines, chemokines, and growth factors alongside oxidative and nitrosative stress biomarkers that are associated with depressive symptoms and MetS (de Melo *et al*., 2017).

## Conclusion

This study illustrates that hsCRP is insufficient as a biomarker for depressive symptoms in patients with MetS, even when individuals exhibit a substantial inflammatory burden. The depressive symptoms in MetS are predominantly associated with atherogenicity (increased ApoB and LDL), insulin resistance (increased TyG index), lower visfatin and adiponectin levels, but also with immune activation indicators (IL-6 and leukocyte number) and platelet aberrations (lower MPV). Elevated hsCRP levels in MetS are significantly correlated with LIPUR indicators indicative of liver dysfunction, uric acid, platelet activation, and immunological activation, a phenomenon seemingly influenced by heightened production of IL-6 and TNF-α. There are no associations between depressive symptoms and either hsCRP or both MetS phenotypes. These results again indicate that combining individuals with and without MetS/obesity to examine the interrelations between depression, hsCRP, and metabolic biomarkers is obsolete.

The identification of two distinct LIPUR subgroups emphasizes the heterogeneity of MetS and reinforces the need for specific assessment of concomitant symptoms and treatment modalities. Individuals in the lower-LIPUR subgroup may necessitate interventions aimed at lifestyle adjustments and pharmacological therapies to treat atherogenicity and insulin sensitivity and improve metabolic health. Patients with the high-LIPUR phenotype may benefit from supplementary targeted therapy designed to mitigate hepatic dysfunction, elevated uric acid synthesis, the activation of platelets, and increased IL-6 and TNF-α production.

## Data Availability

The corresponding author (MM) will provide access to the dataset supporting this study upon receipt of a valid request and the completion of a thorough data review.

## References

[ref1] Akbaraly TN , Ancelin M-L , Jaussent I , Ritchie C , Barberger-Gateau P , Dufouil C , Kivimaki M , Berr C and Ritchie K (2011) Metabolic syndrome and onset of depressive symptoms in the elderly: findings from the three-city study. Diabetes Care 34, 904–909. 21346185 10.2337/dc10-1644 PMC3064049

[ref2] Akbaraly TN , KivimäKi M , Brunner EJ , Chandola T , Marmot MG , Singh-Manoux A and Ferrie JE (2009) Association between metabolic syndrome and depressive symptoms in middle-aged adults: results from the whitehall II study. Diabetes Care 32, 499–504. 19106378 10.2337/dc08-1358 PMC2646036

[ref3] Akinlade K , Adedokun K , Rahamon S and Lasebikan V (2020) Elevated levels of Visfatin and Fetuin-A in patients with major mental disorders. Archives of Basic and Applied Medicine 8, 49–53.

[ref4] Alberti KG , Eckel RH , Grundy SM , Zimmet PZ , Cleeman JI , Donato KA , Fruchart JC , James WP , Loria CM and Smith SC Jr (2009) Harmonizing the metabolic syndrome: a joint interim statement of the international diabetes federation task force on epidemiology and prevention; National Heart, Lung, and Blood Institute; American Heart Association; World Heart Federation; International Atherosclerosis Society; and International Association for the Study of Obesity. Circulation 120, 1640–1645. 19805654 10.1161/CIRCULATIONAHA.109.192644

[ref5] Aldossary KM , Ali LS , Abdallah MS , Bahaa MM , Elmasry TA , Elberri EI , Kotkata FA , El Sabaa RM , Elmorsi YM , Kamel MM , Negm WA , Elberri AI , Hamouda AO , Alrasheed HA , Salahuddin MM , Yasser M and Hamouda MA (2024) Effect of a high dose atorvastatin as added-on therapy on symptoms and serum AMPK/NLRP3 inflammasome and IL-6/STAT3 axes in patients with major depressive disorder: randomized controlled clinical study. Frontiers in Pharmacology 15, 1381523. 38855751 10.3389/fphar.2024.1381523 PMC11157054

[ref6] Alghasham AA and Barakat YA (2008) Serum visfatin and its relation to insulin resistance and inflammation in type 2 diabetic patients with and without macroangiopathy. Saudi Medical Journal 29, 185–192. 18246224

[ref7] Almulla AF , Thipakorn Y , AaA Algon , Tunvirachaisakul C , Al-Hakeim HK and Maes M (2023) Reverse cholesterol transport and lipid peroxidation biomarkers in major depression and bipolar disorder: a systematic review and meta-analysis. Brain Behavior and Immunity 113, 374–388. 37557967 10.1016/j.bbi.2023.08.007

[ref8] Alshammary AF , Alharbi KK , Alshehri NJ , Vennu V and Ali Khan I (2021) Metabolic syndrome and coronary artery disease risk: a meta-analysis of observational studies. International Journal of Environmental Research and Public Health 18, 1773. 33670349 10.3390/ijerph18041773 PMC7918238

[ref9] Anderson G (2025) Reframing polymyalgia pathoetiology, pathophysiology and treatment: role of aging-linked alterations in night-time dampening and resetting by melatonin and cortisol in modulation of local melatonin production via the STAT3 interface with NF-kB dimer composition, with future research and treatment implications. ResearchGate, preprint.

[ref10] Blaha MJ , Rivera JJ , Budoff MJ , Blankstein R , Agatston A , O’leary DH , Cushman M , Lakoski S , Criqui MH , Szklo M , Blumenthal RS and Nasir K (2011) Association between obesity, high-sensitivity C-reactive protein ≥2 mg/L, and subclinical atherosclerosis: implications of JUPITER from the multi-ethnic study of Atherosclerosis. Arteriosclerosis, Thrombosis, and Vascular Biology 31, 1430–1438. 21474823 10.1161/ATVBAHA.111.223768 PMC3130297

[ref11] Bloemer J , Pinky PD , Govindarajulu M , Hong H , Judd R , Amin RH , Moore T , Dhanasekaran M , Reed MN and Suppiramaniam V (2018) Role of adiponectin in central nervous system disorders. Neural Plasticity 2018, 4593530. 30150999 10.1155/2018/4593530 PMC6087588

[ref12] Chirinos DA , Goldberg R , Gellman M , Mendez AJ , Gutt M , Mccalla JR , Llabre MM and Schneiderman N (2013) Leptin and its association with somatic depressive symptoms in patients with the metabolic syndrome. Annals of Behavioral Medicine 46, 31–39. 23436275 10.1007/s12160-013-9479-5 PMC3696025

[ref13] Chirinos DA , Murdock KW , Leroy AS and Fagundes C (2017) Depressive symptom profiles, cardio-metabolic risk and inflammation: results from the MIDUS study. Psychoneuroendocrinology 82, 17–25. 28486177 10.1016/j.psyneuen.2017.04.011 PMC5833295

[ref14] Clarke D , Johnson PWM , Banks RE , Storr M , Kinsey SE , Johnson R , Morgan G , Gordon MY , Illingworth JM , Perren TJ and Selby PJ (1996) Effects of interleukin 6 administration on platelets and haemopoietic progenitor cells in peripheral blood. Cytokine 8, 717–723. 8932983 10.1006/cyto.1996.0095

[ref15] Córdoba-Moreno MO , Santos GC , Muxel SM , Dos Santos-Silva D , Quiles CL , Sousa KDS , Markus RP and Fernandes P (2024) IL-10-induced STAT3/NF-κB crosstalk modulates pineal and extra-pineal melatonin synthesis. Journal of Pineal Research 76, e12923. 37990784 10.1111/jpi.12923

[ref16] Dan K , Gomi S , Inokuchi K , Ogata K , Yamada T , Ohki I , Hasegawa S and Nomura T (2009) Effects of lnterleukin-1 and tumor necrosis factor on megakaryocytopoiesis: mechanism of reactive Thrombocytosis. Acta Haematologica 93, 67–72. 10.1159/000204114 7639054

[ref17] De Melo LGP , Nunes SOV , Anderson G , Vargas HO , Barbosa DS , Galecki P , Carvalho AF and Maes M (2017) Shared metabolic and immune-inflammatory, oxidative and nitrosative stress pathways in the metabolic syndrome and mood disorders. Progress in Neuro-Psychopharmacology and Biological Psychiatry 78, 34–50. 28438472 10.1016/j.pnpbp.2017.04.027

[ref18] Douglas KM , Taylor AJ and O’malley PG (2004) Relationship between depression and C-reactive protein in a screening population. Biopsychosocial Science and Medicine 66, 679–683. 10.1097/01.psy.0000138132.66332.85 15385691

[ref19] Erfani S , Khaksari M , Oryan S , Shamsaei N , Aboutaleb N , Nikbakht F , Jamali-Raeufy N and Gorjipour F (2015) Visfatin reduces hippocampal CA1 cells death and improves learning and memory deficits after transient global ischemia/reperfusion. Neuropeptides 49, 63–68. 25617953 10.1016/j.npep.2014.12.004

[ref20] Fahed G , Aoun L , Bou Zerdan M , Allam S , Bou Zerdan M , Bouferraa Y and Assi HI (2022) Metabolic syndrome: updates on pathophysiology and management in 2021. International Journal of Molecular Sciences 23, 786. 35054972 10.3390/ijms23020786 PMC8775991

[ref21] Foley É.M , Parkinson JT , Mitchell RE , Turner L and Khandaker GM (2023) Peripheral blood cellular immunophenotype in depression: a systematic review and meta-analysis. Molecular Psychiatry 28, 1004–1019. 36577838 10.1038/s41380-022-01919-7 PMC10005954

[ref22] Frühbeck G , Catalán V , Rodríguez A , Ramírez B , Becerril S , Salvador J , Colina I and Gómez-Ambrosi J (2019) Adiponectin-leptin ratio is a functional biomarker of adipose tissue inflammation. Nutrients 11. 10.3390/nu11020454 PMC6412349 30813240

[ref23] Fukuhara A , Matsuda M , Nishizawa M , Segawa K , Tanaka M , Kishimoto K , Matsuki Y , Murakami M , Ichisaka T , Murakami H , Watanabe E , Takagi T , Akiyoshi M , Ohtsubo T , Kihara S , Yamashita S , Makishima M , Funahashi T , Yamanaka S , Hiramatsu R , Matsuzawa Y and Shimomura I (2005) Visfatin: a protein secreted by visceral fat that mimics the effects of insulin. Science 307, 426–430. 15604363 10.1126/science.1097243

[ref24] Furman JL , Soyombo A , Czysz AH , Jha MK , Carmody TJ , Mason BL , Scherer PE and Trivedi MH (2018) Adiponectin moderates antidepressant treatment outcome in the combining medications to enhance depression outcomes randomized clinical trial. Personalized Medicine in Psychiatry 9, 1–7. 30859144 10.1016/j.pmip.2018.05.001 PMC6408148

[ref25] Gardner FH and Bessman JD (1983) Thrombocytopenia due to defective platelet production. Clinics in Haematology 12, 23–38. 6340881

[ref26] Gurka MJ , Vishnu A , Okereke OI , Musani S , Sims M and Deboer MD (2016) Depressive symptoms are associated with worsened severity of the metabolic syndrome in African American women independent of lifestyle factors: a consideration of mechanistic links from the Jackson heart study. Psychoneuroendocrinology 68, 82–90. 26963374 10.1016/j.psyneuen.2016.02.030 PMC5105331

[ref27] Heisey HD , Qualls C , Villareal DT , Segoviano-Escobar MB , Nava MLD , Gatchel JR and Kunik ME (2024) Depressive symptoms are associated with C-reactive protein in older adults with obesity. Journal of Geriatric Psychiatry and Neurology 37, 332–338. 37950647 10.1177/08919887231215041 PMC11087374

[ref28] Hu Y , Dong X and Chen J (2015) Adiponectin and depression: a meta-analysis. Biomedical Reports 3, 38–42. 25469244 10.3892/br.2014.372 PMC4251146

[ref29] Islam S , Islam T , Nahar Z , Shahriar M , Islam SMA , Bhuiyan MA and Islam MR (2022) Altered serum adiponectin and interleukin-8 levels are associated in the pathophysiology of major depressive disorder: a case-control study. PloS One 17, e0276619. 36409748 10.1371/journal.pone.0276619 PMC9678262

[ref30] Ji Y , Wang J , Chen H , Li J and Chen M (2024) Association between hs-CRP and depressive symptoms: a cross-sectional study. Frontiers in Psychiatry 15. 10.3389/fpsyt.2024.1339208 PMC11002220 38596631

[ref31] Jirakran K , Almulla AF , Jaipinta T , Vasupanrajit A , Jansem P , Tunvirachaisakul C , Dzhambazova E , Stoyanov DS and Maes M (2025 *a*) Increased atherogenicity in mood disorders: a systematic review, meta-analysis and meta-regression. Neuroscience & Biobehavioral Reviews 169, 106005. 39793682 10.1016/j.neubiorev.2025.106005

[ref32] Jirakran K , Vasupanrajit A , Tunvirachaisakul C , Almulla AF , Kubera M and Maes M (2025 *b*) Lipid profiles in major depression, both with and without metabolic syndrome: associations with suicidal behaviors and neuroticism. BMC Psychiatry 25, 379. 40234788 10.1186/s12888-025-06734-2 PMC11998271

[ref33] Junqueira AS , Romêo Filho LJ and Junqueira Cde L (2009) Evaluation of the degree of vascular inflammation in patients with metabolic syndrome. Arquivos Brasileiros De Cardiologia 93, 360-6–353–9. 10.1590/s0066-782x2009001000008 19936455

[ref34] Kaser A , Brandacher G , Steurer W , Kaser S , Offner FA , Zoller H , Theurl I , Widder W , Molnar C , Ludwiczek O , Atkins MB , Mier JW and Tilg H (2001) Interleukin-6 stimulates thrombopoiesis through thrombopoietin: role in inflammatory thrombocytosis. Blood 98, 2720–2725. 11675343 10.1182/blood.v98.9.2720

[ref35] Khan A , Leonard D , Defina L , Barlow CE , Willis B and Brown ES (2020) Association between C reactive protein and depression in a population of healthy adults: the cooper center longitudinal study. Journal of Investigative Medicine 68, 1019–1023. 32200354 10.1136/jim-2019-001254

[ref36] Komatsu M , Ohfusa H , Aizawa T and Hashizume K (2007) Adiponectin inversely correlates with high sensitive C-reactive protein and triglycerides, but not with insulin sensitivity, in apparently healthy Japanese men. Endocrine Journal 54, 553–558. 17575367 10.1507/endocrj.k07-032

[ref37] Korniluk A , Koper-Lenkiewicz OM , Kamińska J , Kemona H and Dymicka-Piekarska V (2019) Mean platelet volume (MPV): new perspectives for an old marker in the course and prognosis of inflammatory conditions. Mediators of inflammation 2019, 9213074. 31148950 10.1155/2019/9213074 PMC6501263

[ref38] Kowalska I , Karczewska-Kupczewska M , Adamska A , Nikolajuk A , Otziomek E and Straczkowski M (2013) Serum visfatin is differentially regulated by insulin and free fatty acids in healthy men. The Journal of clinical endocrinology and metabolism 98, E293–7. 23284011 10.1210/jc.2012-2818

[ref39] Krastev D (2020) Linguistic and cross-cultural adaptation of the detlev von zerssen depression scale in a sample of oncological patients. Bulgarian Medicine 10, 36–52.

[ref40] Krogh J , Benros ME , Jørgensen MB , Vesterager L , Elfving B and Nordentoft M (2014) The association between depressive symptoms, cognitive function, and inflammation in major depression. Brain, Behavior, and Immunity 35, 70–76. 24016864 10.1016/j.bbi.2013.08.014

[ref42] Labad J , Price JF , Strachan MWJ , Fowkes FGR , Deary IJ , Seckl JR , Walker BR , Sattar N , Reynolds RM and Investigators OBOTETDS (2012) Leptin levels and depressive symptoms in people with Type 2 diabetes: the Edinburgh Type 2 diabetes study. Biopsychosocial Science and Medicine 74, 39–45. 10.1097/PSY.0b013e31823ba8af 22210236

[ref43] Lee TH , Cheng KK , Hoo RL , Siu PM and Yau SY (2019) The novel perspectives of Adipokines on brain health. International Journal of Molecular Sciences 20. 10.3390/ijms20225638 PMC6887733 31718027

[ref44] Lee YH and Bae SC (2018) Circulating adiponectin and visfatin levels in rheumatoid arthritis and their correlation with disease activity: a meta-analysis. International Journal of Rheumatic Diseases 21, 664–672. 28205390 10.1111/1756-185X.13038

[ref45] Liu J , Guo M , Zhang D , Cheng SY , Liu M , Ding J , Scherer PE , Liu F and Lu XY (2012) Adiponectin is critical in determining susceptibility to depressive behaviors and has antidepressant-like activity. Proceedings of the National Academy of Sciences 109, 12248–12253. 10.1073/pnas.1202835109 PMC3409774 22778410

[ref46] Maes M (2025) The concepts of “inflammatory depression” and “immune-mediated depression” as defined by the C-reactive protein criterion are inaccurate. ResearchGate, preprint.

[ref47] Maes M , Almulla AF and Al-Hakeim HK (2023) Increased insulin resistance is associated with depressive symptoms due to long COVID. Brazilian Journal of Psychiatry 45, 380–381. 37127292 10.47626/1516-4446-2023-0048 PMC10668318

[ref85] Maes M , Almulla AF , You Z and Zhang Y (2025a) Neuroimmune, metabolic and oxidative stress pathways in major depressive disorder. Nat Rev Neurol. 10.1038/s41582-025-01116-4 40659853

[ref48] Maes M , Jirakran K , Semeão LDO , Michelin AP , Matsumoto AK , Brinholi FF , Barbosa DS , Tivirachaisakul C , Almulla AF , Stoyanov D and Zhang Y (2025b) Key factors underpinning neuroimmune-metabolic-oxidative (NIMETOX) major depression in outpatients: paraoxonase 1 activity, reverse cholesterol transport, increased atherogenicity, protein oxidation, and differently expressed cytokine networks. medRxiv, 2025.03.02.25323183. 40929711

[ref49] Maes M , Jirakran K , Vasupanrajit A , Zhou B , Tunvirachaisakul C and Almulla AF (2024) Major depressive disorder, neuroticism, suicidal behaviors, and depression severity are associated with cytokine networks and their intricate interactions with metabolic syndrome. Journal of Psychosomatic Research 187, 111951. 39413534 10.1016/j.jpsychores.2024.111951

[ref86] Maes M , Jirakran K , Vasupanrajit A , Zhou B , Tunvirachaisakul C , Stoyanov DS and Almulla AF (2024) Are abnormalities in lipid metabolism, together with adverse childhood experiences, the silent causes of immune-linked neurotoxicity in major depression? Neuro Endocrinol Lett,45, 393-408. 39732467

[ref51] Maes M , Meltzer HY , Bosmans E , Bergmans R , Vandoolaeghe E , Ranjan R and Desnyder R (1995) Increased plasma concentrations of interleukin-6, soluble interleukin-6, soluble interleukin-2 and transferrin receptor in major depression. Journal of Affective Disorders 34, 301–309. 8550956 10.1016/0165-0327(95)00028-l

[ref52] Maes M , Scharpe S , Meltzer HY , Bosmans E , Suy E , Calabrese J and Cosyns P (1993a) Relationships between interleukin-6 activity, acute phase proteins, and function of the hypothalamic-pituitary-adrenal axis in severe depression. Psychiatry Research 49, 11–27. 7511248 10.1016/0165-1781(93)90027-e

[ref53] Maes M , Scharpé S , Meltzer HY and Cosyns P (1993b) Relationships between increased haptoglobin plasma levels and activation of cell-mediated immunity in depression. Biological Psychiatry 34, 690–701. 8292673 10.1016/0006-3223(93)90042-c

[ref55] Mclester CN , Nickerson BS , Kliszczewicz BM and Mclester JR (2020) Reliability and agreement of various inBody body composition analyzers as compared to dual-energy X-ray absorptiometry in healthy men and women. Journal of Clinical Densitometry 23, 443–450. 30472111 10.1016/j.jocd.2018.10.008

[ref56] Mirhafez SR , Ebrahimi M , Saberi Karimian M , Avan A , Tayefi M , Heidari-Bakavoli A , Parizadeh MR , Moohebati M , Azarpazhooh MR , Esmaily H , Nematy M , Safarian M , Ferns GA and Ghayour-Mobarhan M (2016) Serum high-sensitivity C-reactive protein as a biomarker in patients with metabolic syndrome: evidence-based study with 7284 subjects. European Journal of Clinical Nutrition 70, 1298–1304. 27460263 10.1038/ejcn.2016.111

[ref57] Mobeen A , Joshi S , Fatima F , Bhargav A , Arif Y , Faruq M and Ramachandran S (2025) NF-κB signaling is the major inflammatory pathway for inducing insulin resistance. 3 Biotech 15, 47. 10.1007/s13205-024-04202-4 PMC11747027 39845928

[ref58] Mommersteeg PMC , Meeuwis SH , Denollet J , Widdershoven JW , Aarnoudse W , Westerhuis BLWJJM and Kop WJ (2014) C-reactive protein and fibrinogen in non-obstructive coronary artery disease as related to depressive symptoms and anxiety: findings from the TweeSteden Mild Stenosis Study (TWIST). Journal of Psychosomatic Research 77, 426–429. 25307791 10.1016/j.jpsychores.2014.09.020

[ref59] Moraes JB , Maes M , Barbosa DS , Ferrari TZ , Uehara MKS , Carvalho AF and Nunes SOV (2017) Elevated C-reactive protein levels in women with bipolar disorder may be explained by a history of childhood trauma, especially sexual abuse, body mass index and age. CNS & Neurological Disorders - Drug Targets 16, 514–521. 28403800 10.2174/1871527316666170407151514

[ref60] Nolan PB , Carrick-Ranson G , Stinear JW , Reading SA and Dalleck LC (2017) Prevalence of metabolic syndrome and metabolic syndrome components in young adults: a pooled analysis. Preventive Medicine Reports 7, 211–215. 28794957 10.1016/j.pmedr.2017.07.004 PMC5540707

[ref61] Ohashi K , Ouchi N and Matsuzawa Y (2012) Anti-inflammatory and anti-atherogenic properties of adiponectin. Biochimie 94, 2137–2142. 22713764 10.1016/j.biochi.2012.06.008

[ref62] Osimo EF , Pillinger T , Rodriguez IM , Khandaker GM , Pariante CM and Howes OD (2020) Inflammatory markers in depression: a meta-analysis of mean differences and variability in 5,166 patients and 5,083 controls. Brain, Behavior, and Immunity 87, 901–909. 32113908 10.1016/j.bbi.2020.02.010 PMC7327519

[ref63] Öztürk A , Şahan E , Mirçik AB , Deveci E , Yilmaz O and Kirpinar I (2019) Mean platelet volume and neutrophil to lymphocyte ratio decrease in patients with depression with antidepressant treatment. Archives of Clinical Psychiatry (São Paulo) 46, 9–13.

[ref64] Pfeffer KD , Huecksteadt TP and Hoidal JR (1994) Xanthine dehydrogenase and xanthine oxidase activity and gene expression in renal epithelial cells. Cytokine and steroid regulation.. Journal of immunology (Baltimore, Md. : 1950) 153, 1789–1797. 8046245

[ref65] Pulkki-Råback L , Elovainio M , Kivimäki M , Mattsson N , Raitakari OT , Puttonen S , Marniemi J , Viikari JS and Keltikangas-Järvinen L (2009) Depressive symptoms and the metabolic syndrome in childhood and adulthood: a prospective cohort study. Health Psychology 28, 108. 19210024 10.1037/a0012646 PMC3166561

[ref66] Saadoun MM , NaE Nosair , Abdel-Azeez HA , Sharaf SM and Ahmed MH (2021) Serum visfatin as a diagnostic marker of active inflammatory bowel disease. Journal of Gastrointestinal and Liver Diseases 30, 339–345. 34551033 10.15403/jgld-3504

[ref67] Schmidt-Arras D and Rose-John S (2016) IL-6 pathway in the liver: from physiopathology to therapy. Journal of Hepatology 64, 1403–1415. 26867490 10.1016/j.jhep.2016.02.004

[ref68] Shamsuzzaman ASM , Winnicki M , Wolk R , Svatikova A , Phillips BG , Davison DE , Berger PB and Somers VK (2004) Independent association between plasma leptin and C-reactive protein in healthy humans. Circulation 109, 2181–2185. 15117839 10.1161/01.CIR.0000127960.28627.75

[ref69] Shih Y-L , Lin Y and Chen J-Y (2022) The association between high-sensitivity C-reactive protein and metabolic syndrome in an elderly population aged 50 and older in a community receiving primary health care in Taiwan. International Journal of Environmental Research and Public Health 19, 13111. 36293692 10.3390/ijerph192013111 PMC9603035

[ref54] Sigdel M , Kumar A , Gyawali P , Shrestha R , Tuladhar ET , Jha B (2014) Association of high sensitivity C-reactive protein with the components of metabolic syndrome in diabetic and non-diabetic individuals. Journal of Clinical and Diagnostic Research 8, CC11–CC13. 10.7860/JCDR/2014/8085.4522 PMC4129304 25120975

[ref70] Stofkova A (2010) Resistin and visfatin: regulators of insulin sensitivity, inflammation and immunity. Endocrine Regulations 44, 25–36. 20151765 10.4149/endo_2010_01_25

[ref71] Suttajit S and Pilakanta S (2013) Prevalence of metabolic syndrome and its association with depression in patients with schizophrenia. Neuropsychiatric Disease and Treatment 9, 941–946. 23882141 10.2147/NDT.S47450 PMC3709830

[ref72] Thomas MR and Storey RF (2015) The role of platelets in inflammation. Thrombosis and Haemostasis 114, 449–458. 26293514 10.1160/TH14-12-1067

[ref73] Timpson NJ , Nordestgaard BG , Harbord RM , Zacho J , Frayling TM , Tybjærg-Hansen A and Smith GD (2011) C-reactive protein levels and body mass index: elucidating direction of causation through reciprocal mendelian randomization. International Journal of Obesity 35, 300–308. 20714329 10.1038/ijo.2010.137 PMC4783860

[ref74] Toth B , Yokoyama Y , Schwacha MG , George RL , Rue LW , Bland KI and Chaudry IH (2004) Insights into the role of interleukin-6 in the induction of hepatic injury after trauma-hemorrhagic shock. Journal of Applied Physiology 97, 2184–2189. 15298985 10.1152/japplphysiol.00499.2004

[ref75] Tsutani H , Yoshio N and Ueda T (2000) Interleukin 6 reduces serum urate concentrations. Journal of Rheumatology 27, 554. 10685834

[ref76] Tully PJ , Baumeister H , Bengel J , Jenkins A , Januszewski A , Martin S and Wittert GA (2015) The longitudinal association between inflammation and incident depressive symptoms in men: the effects of hs-CRP are independent of abdominal obesity and metabolic disturbances. Physiology & Behavior 139, 328–335. 25460540 10.1016/j.physbeh.2014.11.058

[ref77] Villarreal-Zegarra D and Bernabe-Ortiz A (2020) Association between arterial hypertension and depressive symptoms: results from population-based surveys in Peru. Asia-Pacific Psychiatry 12, e12385. 32119760 10.1111/appy.12385

[ref78] Vrints C , Andreotti F , Koskinas KC , Rossello X , Adamo M , Ainslie J , Banning AP , Budaj A , Buechel RR , Chiariello GA , Chieffo A , Christodorescu RM , Deaton C , Doenst T , Jones HW , Kunadian V , Mehilli J , Milojevic M , Piek JJ , Pugliese F , Rubboli A , Semb AG , Senior R , Ten Berg JM , Van Belle E , Van Craenenbroeck EM , Vidal-Perez R , Winther S and Group ESCSD (2024 2024) ESC guidelines for the management of chronic coronary syndromes: developed by the task force for the management of chronic coronary syndromes of the European society of cardiology (ESC) endorsed by the European association for cardio-thoracic surgery (EACTS). European Heart Journal 45, 3415–3537. 39210710 10.1093/eurheartj/ehae177

[ref79] Wang Q , Wang S , Zhuang Z , Wu X , Gao H , Zhang T , Zou G , Ge X and Liu Y (2025) Identification of potential crucial genes and mechanisms associated with metabolically unhealthy obesity based on the gene expression profile. Frontiers in Genetics 16, 1540721. 40376303 10.3389/fgene.2025.1540721 PMC12078199

[ref80] Wessa C , Janssens J , Coppens V , El Abdellati K , Vergaelen E , Van Den Ameele S , Baeken C , Zeeuws D , Milaneschi Y , Lamers F , Penninx B , Claes S , Morrens M and De Picker L (2024) Efficacy of inflammation-based stratification for add-on celecoxib or minocycline in major depressive disorder: protocol of the INSTA-MD double-blind placebo-controlled randomised clinical trial. Brain, Behavior, & Immunity - Health 41, 100871. 10.1016/j.bbih.2024.100871 PMC11440344 39350954

[ref81] World Health O (2011) Waist circumference and waist-hip ratio : report of a WHO expert consultation, Geneva, 8-11 December 2008. World Health Organization.

[ref82] Xu K , Wang M , Wang H , Zhao S , Tu D , Gong X , Li W , Liu X , Zhong L , Chen J and Xie P (2024) HMGB1/STAT3/p65 axis drives microglial activation and autophagy exert a crucial role in chronic stress-induced major depressive disorder. Journal of Advanced Research 59, 79–96. 37321346 10.1016/j.jare.2023.06.003 PMC11081938

[ref83] Zelber-Sagi S , Toker S , Armon G , Melamed S , Berliner S , Shapira I , Halpern Z , Santo E and Shibolet O (2013) Elevated alanine aminotransferase independently predicts new onset of depression in employees undergoing health screening examinations. Psychological Medicine 43, 2603–2613. 23522007 10.1017/S0033291713000500

[ref84] Zhang Z , Wang P , Xiong Q , Xu S , Kang D , He Z , Yao C and Jian G (2024) Advancements in the study of IL-6 and its receptors in the pathogenesis of gout. Cytokine 182, 156705. 39053079 10.1016/j.cyto.2024.156705

